# ADP-ribosyl-N_3_: A Versatile Precursor for Divergent Syntheses of ADP-ribosylated Compounds

**DOI:** 10.3390/molecules22081346

**Published:** 2017-08-14

**Authors:** Lingjun Li, Qianqian Li, Shengqiang Ding, Pengyang Xin, Yuqin Zhang, Shenlong Huang, Guisheng Zhang

**Affiliations:** Collaborative Innovation Center of Henan Province for Green Manufacturing of Fine Chemicals, Key Laboratory of Green Chemical Media and Reactions, Ministry of Education, School of Chemistry and Chemical Engineering Henan Normal University, Xinxiang 453007, Henan, China; 15225989055@163.com (Q.L.); sqding163@163.com (S.D.); pyxin27@163.com (P.X.); zyq18143978269@163.com (Y.Z.); 18236132813@163.com (S.H.);

**Keywords:** nucleotides, ADP-ribosylated compounds, divergent synthesis

## Abstract

Adenosine diphosphate-ribose (ADP-ribose) and its derivatives play important roles in a series of complex physiological procedures. The design and synthesis of artificial ADP-ribosylated compounds is an efficient way to develop valuable chemical biology tools and discover new drug candidates. However, the synthesis of ADP-ribosylated compounds is currently difficult due to structural complexity, easily broken pyrophosphate bond and high hydrophilicity. In this paper, ADP-ribosyl-N_3_ was designed and synthesized for the first time. With ADP-ribosyl-N_3_ as the key precursor, a divergent post-modification strategy was developed to prepare structurally diverse ADP-ribosylated compounds including novel nucleotides and peptides bearing ADP-ribosyl moieties.

## 1. Introduction

**Figure 1:**
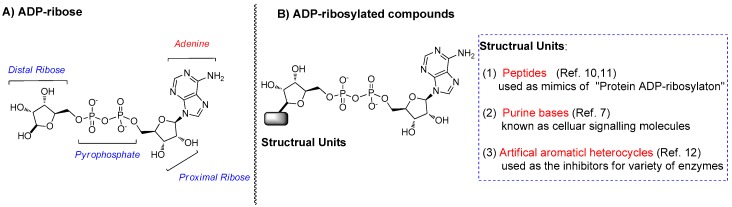
ADP-ribose and ADP-ribosylated compounds. (**A**) Structure of ADP-ribose; (**B**) Structurally and functionally diverse ADP-ribosylated compounds.

Adenosine diphosphate-ribose (ADP-ribose) that was shown in Figure 1A and its derivatives have indicated a series of physiological functions. For example, ADP-ribosylation that involves the enzymatic transfer of ADP-ribose from nicotinamide adenine dinucleotide (NAD^+^) to a side chain of amino acid, can dynamically control physiological procedures of target proteins, and is associated with DNA damage, apoptosis, and gene regulation [1,2]. On the other hand, ADP-ribose is the natural receptor of acetyl groups in sirtuin-catalyzing deacetylations of acetyllysine residues. Compounds bearing ADP-ribosyl moieties are thus sought as inhibitor candidates of human sirtuin family (SIRT1–7) which are implicated in essential cellular processes such as transcriptional control, cell cycle progression, and aging [3,4]. Besides, ADP-ribose itself, cyclic ADP-ribose and other endogenous dinucleotides containing ADP-ribosyl moieties have been identified to regulate physiology and pathology of calcium-controlled cell functions in the past decades [5,6,7].

**Scheme 1:**
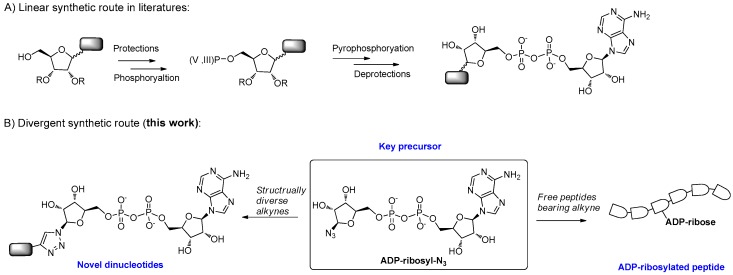
Linear synthetic routes or divergent routes for the syntheses of ADP-ribosylated compounds. (**A**) The linear synthetic route for preparation of ADP-ribosylated compounds in literature; (**B**) The divergent synthetic route for preparation of ADP-ribosylated compounds in this work.

Design and synthesis of artificial ADP-ribosylated compounds (Figure 1B) are important for the investigations of physiological roles of ADP-ribose and discovery of new drug candidates [8,9,10,11,12,13]. Traditional synthetic routes for these ADP-ribosylated compounds is generally linear, which start with the base-modified nucleosides as starting materials, followed with phosphoryaltion and pyrophosphoryation (Scheme 1A) [11,12]. The linear synthetic route requires repeating phosphorylation and pyrophosphoryation procedure in harsh reaction conditions for each ADP-ribosylated compound syntheses. In addition, the corresponding phosphate products have high polarity and high solubility in water, which makes the related isolation and workup procedure tedium and low efficient. Therefore, development of new synthetic route allowing more efficient access to structurally diverse ADP-ribosylated compounds is in great need but challenging.

Divergent strategy has recently attracted much attention for synthesizing natural products and artificial molecules in an efficient manner [14,15,16]. Using a precursor or intermediate bearing functional groups that allows potentially chemoselective transformations on the specific sites of natural products, divergent preparation of diverse compounds could be conveniently realized through post-modifications without interrupting other delicate parts of the parent compounds. In a divergent route, deliberately designed precursors are usually vital for the following diverse post-modifications. Herein, ADP-ribosyl-N_3_ was designed and synthesized. As a readily accessible and easy-to-handle precursor, ADP-ribosyl-N_3_ enabled a divergent strategy to synthesize ADP-ribosylated compounds, which was further developed and utilized successfully in preparation of structurally novel dinucleotides and conjugation of free peptides (Scheme 1B).

## 2. Results and Discussion

### 2.1. Synthesis of ADP-ribosyl-N_3_

**Figure 2:**
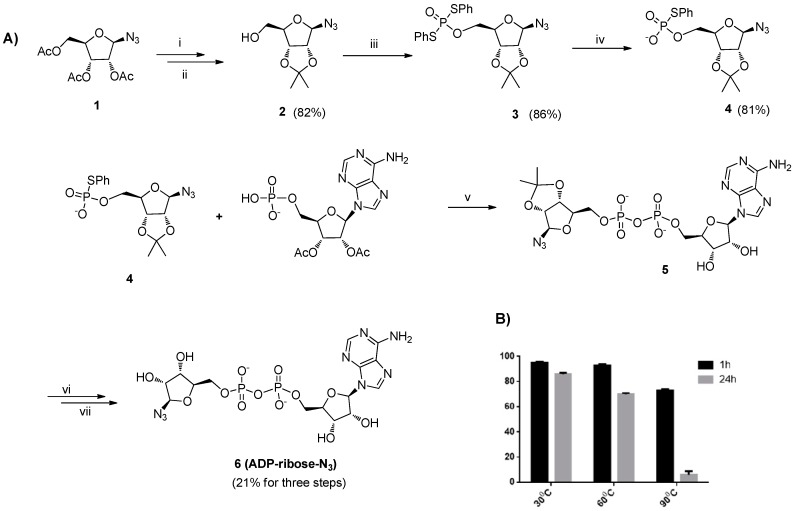
(**A**) Synthesis route of ADP-ribosyl-N_3_. Reagent and reaction conditions: (i) I_2_, MeOH; (ii) H^+^/acetone; (iii) (PhS)_2_POCl, TPSCl, tetrazole, Py; (iv) H_3_PO_2__,_ Py; (v) I_2_, MS, Py; (vi) 30% NH_4_OH; (vii) 50% HCOOH; (**B**) The solution stability of ADP-ribosyl-N_3_. Conditions: 1 mM aqueous solution of ADP-ribosyl-N_3_ was heated at given temperatures, and the resultant mixtures were detected by HPLC.

Considering the feasible synthesis, adequate stability and easily functionalization for post modifications, ADP-ribosyl-N_3_ was designed as a precursor for divergent syntheses of ADP-ribosylated compounds. Synthesis of ADP-ribosyl-N_3_ was realized by the following procedure. Firstly, we prepared phosphorylated ribosyl azide **4** (see **4** in Figure 2) as one of donors for intermolecular pryophosphorylation reaction. Acetylated ribosyl azide was used as starting material for the synthesis of compound **4**. After transferring of protection groups, the phosphorylation on 5′-hydroxyl group of compound **2** proceeded effectively with (PhS)_2_POCl to give compound **3** with 86% yield. The following deprotection of PhS- group can be completed in the presence of H_3_PO_2_, and compound **4** was obtained in 81% yield. Then, pryophosphorylation reaction between **4** and acetylated adenosine monophosphate (AMP) was conducted in dry pyridine with I_2_ as catalyst. The deprotection of acetyl and ketal groups was finally completed by treatment of reaction mixture with 30% NH_4_OH and then 50% HCOOH. The target compound **6** (ADP-ribosyl-N_3_) could be given through the purification by HPLC. The structure of ADP-ribosyl-N_3_ was identified by ^1^H-NMR, ^31^P-NMR, ^1^H-^1^H COSY and high resolution mass spectrum (Supporting Information). The yield of three steps from **4** to **6** was 21%, indicating a higher efficiency compared with other methods for pryophosphorylation like the widely-used *N*,*N*-diisopropylcarbodiimide (DIC) system [11]. Total yield for the whole synthetic route was 12% from compound **2**, twice HPLC purification procedures were used, and no anomerizations for glucosidic bonds were observed in all steps.

In order to further develop a versatile platform for divergent preparations of ADP-ribosylated compounds, we investigated the stability of compound **6**. Compound **6** was obtained after lyophilization as the triethylamine salt of ADP-ribosyl-N_3_. This salt was a stable white powder. No degenerations could be detected after exposing it at room temperature under open air conditions for 24 h. The solution stability of compound **6** was investigated through heating its aqueous solution at different temperatures (Figure 2B). No dramatic degenerations of ADP-ribosyl-N_3_ were found after 24 h at 30 °C. After incubating of 1 mM compound **6** at 90 °C for 1 h, there was still 78% ADP-ribosyl-N_3_ that could be recovered by HPLC. Considering readily accessible and cheap starting materials, moderate total yield, high stability and ease to restore, this ADP-ribosyl-N_3_ salt offered a desirable synthetic precursor for divergent preparations of various ADP-ribosylated compounds.

### 2.2. Preparation of Dinucleotides with ADP-ribosyl-N_3_

We then investigated the potential of ADP-ribosyl-N_3_ for the preparations of structurally novel dinucleotides. 1,2,3-triazolyl moiety is a bioisostere of natural heterocycle, which has indicated an interesting perspective for designs of drug candidates with different biological activities [17,18,19]. Building of variant substituted-1,2,3-triazolyl moieties on the bistal ribose of ADP-ribose was expected to complete by the copper-catalyzed alkyne and azide cycloaddition (CuAAC) reaction between ADP-ribosyl-N_3_ and different terminal alkynes. Due to the hydrophility of ADP-ribosyl-N_3_, aqueous conditions that contain CuSO_4_ as copper resource and sodium ascorbate as reducing agent was firstly chosen for CuAAC reactions. However, in presence of 10 mol% CuSO_4_ and 5.0 equivalent of sodium ascorbate, the reaction between ADP-ribosyl-N_3_ and phenylacetylene proceeded sluggish with an unexpected low yield (<5%) after 24 h. Attempts to improve the reaction efficiency through addition of tris[(1-benzyl-1*H*-1,2,3-triazol-4-yl)methyl]amine (TBTA) or tris(3-hydroxypropyltriazolylmethyl)amine (THPTA) as ligands were unsuccessful. Then a wide investigation of a panel of copper catalytic system on our model reactions was conducted with various solvents under different temperatures (Table 1). CuSO_4_/Cu(0) (copper sheet) was finally found as the most effective one. In presence of CuSO_4_/Cu(0) catalytic system, reaction between ADP-ribosyl-N_3_ and phenylacetylene was accelerated dramatically, and product **7a** could be obtained in 91% yields after 12 h at room temperature. A possible reason of this phenomena was that the formation of complex between Cu(II) and pyrophosphate of ADP-ribosyl-N_3_ might depress generations of catalytic copper species for CuAAC reaction [20]. When the system of CuSO_4_/Cu(0) (copper sheet) was used, there is a plenty of copper resource from copper sheet in reaction mixture, and thus catalytic copper species could be provided enough to promote the following cycloaddition reaction effectively.

**Figure 3:**
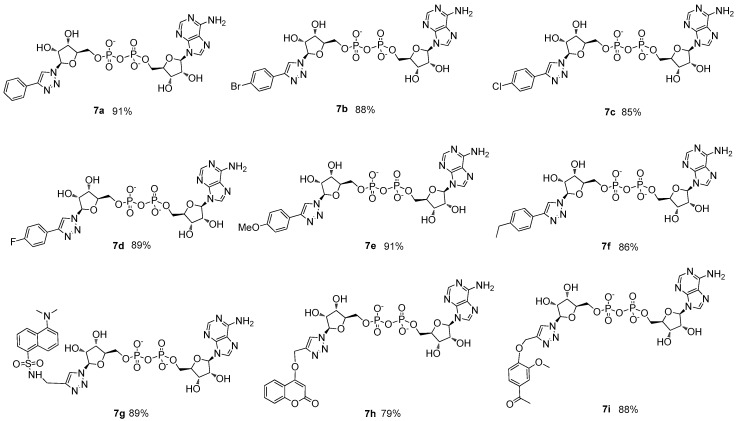
ADP-ribosylated compounds synthesized via divergent strategy ^(^^1,^^2)^. ^1^ ADP-ribosyl-N_3_ (0.01 mmol), terminal alkynes (0.012 mmol), 10 mol % CuSO_4_ and Cu(0) (copper sheet) were added in TEAB buffer (1 mL) and the reaction was stirred for 24 h at room temperature; ^2^ Isolated yields by semiprepartive HPLC.

A survey of reactions between ADP-ribosyl-N_3_ and a variety of alkynes was shown in Figure 3. Aromatic alkynes containing electron-withdrawing Cl-, Br-, F-, and electron-donating MeO-, Et- reacted with ADP-ribosyl-N_3_ to form the dinucleotides (compounds **7b** to **7f** in Figure 3) in very good yields from 85% to 91%. Alkyl alkynes could also proceed smoothly with 79% and 89% yield under the optimized conditions. Besides, other functionalized alkynes containing fluorescent or bioconjugation groups that were used widely in bio-labeling also underwent in the current conditions to form desirable products (compounds **7g**, **7i** in Figure 3).

### 2.3. Conjugation of ADP-ribosyl-N_3_ to Free Peptide

**Scheme 2:**
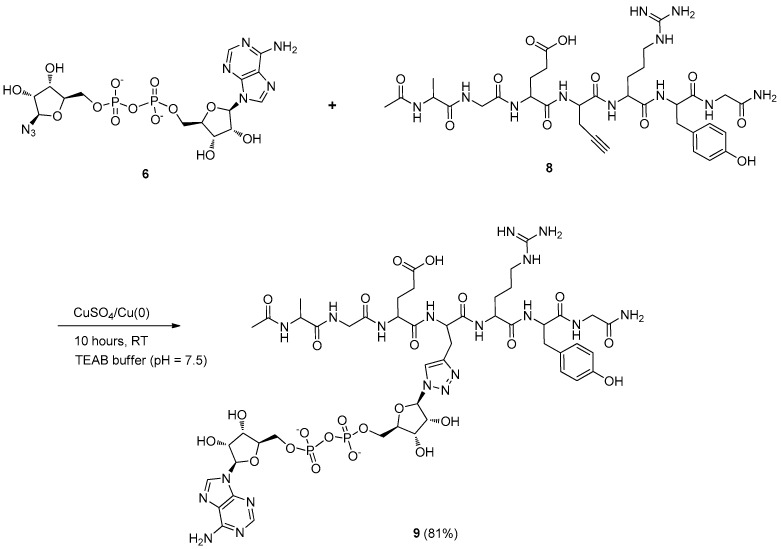
Conjugation of ADP-ribosyl moiety to free peptide with ADP-ribosyl-N_3_ as a building block.

Inspired with successful applications of ADP-ribosyl-N_3_ for the preparations of novel dinucleotide molecules, we next attempted to explore the use of ADP-ribosyl-N_3_ in conjugation of ADP-ribosyl moiety to free peptides. ADP-ribosylated peptides have provided a valuable tool for the studies of protein ADP-ribosylation [10,21]. However, incorporations of ADP-ribosyl moieties to peptides are still challenging. Current methods involved multi-step reactions, low yields, or suffered from the unstable linkages. For example, the method for conjugation of ADP-ribose into peptides was implemented through pryophosphorylation reactions between ribosylated peptide phosphomonoesters and adenosine phosphates [11]. In such synthetic strategy, the one-resin formation of phosphate was a less efficient step. Another method that used oxime ligation for post conjugation of ADP-ribosylated into peptides is concise, but suffers from the generation of ring-opened products and isomers [10]. Herein, we expressed the feasibility of ADP-ribosyl-N_3_ as a effective building block for conjugation of ADP-ribosyl moiety into the free peptide that contain terminal alkyne groups. In our method, connection reaction of ADP-ribosyl-N_3_ and the peptide **8** proceeded efficiently in presence of 75 μM CuSO_4_ with Cu(0) as reducing reagent in triethylammonium bicarbonate (TEAB) buffer (pH = 7.5) (Scheme 2). Target compound **9** was obtained after 10 h at room temperature with 81% isolated yield. Additionally, with 1,2,3-triazole as the linker of ADP-ribosyl and peptide, distal ribose in compound **9** was intact without detectable open-ring products. ADP-ribosyl-N_3_ thus may offer a promising building block for developing alternative conjugation methods of ADP-ribosylated peptide.

## 3. Materials and Methods

### 3.1. General Information and Materials

Solvents were dried using traditional methods, and freshly distilled prior to use. Reactions were monitored by thin-layer chromatography (TLC) on silica gel GF254-precoated plates and RP-HPLC (Agilent Technologies, Santa Clara, CA, USA). Compounds were detected under UV light and/or visualized by phosphomolybdic acid in ethanol solution. Solvents were evaporated under reduced pressure and below 50 °C. Mass spectra were obtained on Bruker APEX (Bruker Daltonics, Billerica, MA, USA). High-resolution MS (HRMS) were performed with Bruker BIFLEX III (Bruker Daltonics, Billerica, MA, USA) and Bruker APEX II (Bruker Daltonics, Billerica, MA, USA). ^1^H-NMR and ^13^C-NMR spectra were recorded on a BrukerAV400 (Bruker Biospin, Ettlingen, Germany) spectrometer or Bruker Avance III-HD 600 NMR Spectrometer (Bruker Biospin, Ettlingen, Germany) using TMS as internal standard. Chemical shifts are reported in parts per million and coupling constants quoted in Hz. Alkyne (a, b, c, d, e, f) CuSO_4_·5H_2_O, Sodium ascorbate and copper sheet from Alfa Aesar (Tianjin, China). Peptide **8** was purchased from GL Biochem (Shanghai, China) Ltd. Starting materials and copies of NMR spectrum of compound **6**, **7a** to **7i** and **9** were put in the Supplementary Materials of this paper.

### 3.2. Synthesis of Compound ***2***

130 mg of compound **1** was dissolved in 4 mL of anhydrous methanol, 50 mg of dry iodine solid was added and the resulted mixture was stirred at room temperature, the reaction process was detected by TLC. After 5 h, the reaction was completed. Then, 0.5 mL of 1 M NaHSO_4_ solution was added to the reaction mixture to adjust the pH to 7.5. After the evaporation of solvents, the residue was washed with methanol for three times. The combined solution was condensed by rotary evaporator to get light yellow syrup, which was further dried under vacuum overnight to give the deacetylated intermediate. The dry deacetylated intermediate was dissolved in 5 mL of dry acetone, and 1 mL of dry 2,2-dimethylpropane and 3 mg of strong-acid cation exchange resin were added. The reaction was detected by TLC, and a new compound was formed after 5 h. Sodium bicarbonate was added to adjust the pH of the reaction solution to 7.0. The solid acid and the excess salt were removed by filtration. After purification by flash chromatography on silica gel (eluent: ethyl acetate-petroleum (b.p. 60–90 °C), compound **2** was obtained in the yield of 82% (76.15 mg) (for two steps) as a colorless liquid. ^1^H-NMR: (300 MHz, CDCl_3_), δ 5.11 (d, *J* = 6 Hz, 1H), 5.54–4.50 (m, 1H), 4.35–4.31 (m, 1H), 3.89–3.84 (dd, *J*_1_ = 10 Hz, *J*_2_ = 3.6 Hz, 1H), 3.76–3.70 (dd, *J*_1_ = 10 Hz, *J*_2_ = 3.6 Hz, 1H), 3.45 (m, 1H), 2.56–2.53 (d, 1H), 1.56, 1.38 (s, each 3H). IR 2010 m^−1^.

### 3.3. Synthesis of Compound ***3***

38 mg of compound **2** was dissolved in 5 mL of dry pyridine and TPSCl (106 mg), (PhS)_2_POCl (167 mg) and 31 mg of tetrazole were added sequentially. The reaction process was detected by TLC, the reaction was completed after 48 h at room temperature. After the purification by flash chromatography on silica gel (eluent: ethyl acetate-petroleum (b.p. 60–90 °C), compound **3** was given in the yield of 86% (145.86 mg). ^1^H-NMR: (300 MHz, CDCl_3_), δ 7.62–7.26 (m, 10H), 5.23–5.20 (d, *J* =7.5 Hz, 1H), 4.56–4.49 (m, 1H), 4.47–4.46 (m, 1H), 4.29–4.26 (m, 1H), 3.79–3.74 (dd, *J*_1_ = 12.0 Hz, *J*_2_ = 2.5 Hz, 1H), 3.71–3.66 (dd, *J*_1_ = 12.5, *J*_2_ = 2.5 Hz, 1H), 1.61, 1.32 (s, each 3H), ^31^P (81 Hz, decoupled with 1H), δ 51.8.

### 3.4. Synthesis of Compound ***4***

Dissolving 13 mg of compound **3** to 1 mL of dry pyridine, and then 56 μL of dry H_2_PO_4_ and 76 μL of triethylamine were added and stirred at room temperature. The reaction was completed after 12 h. And then the pyridine was evaporated, and the residue was partitioned between CHCl_3_ and H_2_O. The aqueous layer was evaporated and the residue was dissolved in 1 mL of methanol. After the purification by flash chromatography on silica gel (eluent: methanol-dichloromethane), compound **4** was given in the yield of 81% (95.21 mg). ^1^H-NMR (500 MHz, CDCl_3_), δ 7.73–7.18 (m, 5H), 5.17–5.15 (d, *J* = 8 Hz, 1H), 4.86–4.84 (m, 1H), 4.32–4.29 (m, 1H), 4.25–4.24 (m, 1H), 3.77–3.74 (dd, *J*_1_ = 12.5 Hz, *J*_2_ = 2.5 Hz), 3.68–3.63 (dd, *J*_1_ = 12.5 Hz, *J*_2_ = 2.5 Hz, 1H), 1.47, 1.26 (s, each 3H), ^31^P-NMR (81 Hz, decoupled with 1H), δ 13.9 (s). HRMS (ESI) *m*/*z* calculate for ([M + H]^+^): C_14_H_18_N_3_O_6_PS^+^: 387.0727, Found: 387.0731.

### 3.5. Synthesis of Compound ***6***

The activated 3 Å molecular sieve (2.0 g) and the molecular iodine 83 mg were added in a round bottom flask, and then 50 mL of anhydrous pyridine was added. The compound **4** (10 mg) was dissolved in 5 mL of anhydrous pyridine at room temperature, and then was injected into the reaction flask within 1.5 h. After the completion of injection, the mixture was stirred for another 2 h. The pyridine was removed with rotary evaporator, and the residue was partitioned between CHCl_3_ and H_2_O. The aqueous layer was evaporated and the residue was dissolved in TEAB buffer (1.0 mL, 0.1 M, pH 7.5), which was applied to a semipreparative C18 reversed-phase column (9.4 mm × 250 mm). The column was eluted using a linear gradient of 0–80% CH_3_CN in TEAB buffer (0.1 M, pH 7.5) over 50 min. After lyophilization, compound **5** was obtained as light-yellow syrup. A solution of **5** in 50% HCOOH (1.5 mL) was stirred for 2 h and then evaporated under reduced pressure. The residue was resolved in 30% aqueous ammonia, stirred for 2 h. After evaporation of solvent, compound **6** was given as crude product. The further purification of the crude product was performed by HPLC on the semipreparative C18 reversed-phase column (9.4 mm × 250 mm) eluted with a linear gradient of 0–65% CH_3_CN in TEAB buffer (0.1 M, pH 7.5) to give the target compound **6** in the yield of 21% (30.14 mg) (for three steps). ^1^H-NMR (400 MHz, D_2_O): δ 8.42 (s, 1H), 8.13 (s, 1H), 6.03 (s, 1H), 5.19 (s, 1H), 4.44 (s, 1H), 4.29 (s, 1H), 4.16–4.14 (m, 4H), 4.04–4.02 (m, 2H), 3.85 (s, 1H), 3.83(S, 1H). ^13^C-NMR (D_2_O, 150 MHz): δ 155.5, 152.9, 149.2, 139.9, 118.6, 94.5, 86.8, 83.8, 82.3, 74.5, 74.3, 70.3, 70.2, 65.6, 65.1, 58.9. ^31^P-NMR (162 MHz, D_2_O): δ −11.57. HRMS (ESI) *m*/*z* calculate for ([M + H]^+^): C_15_H_23_N_8_O_13_P_2_^+^: 585.0727, Found: 585.0856.

### 3.6. Synthesis of Compounds ***7a*** to ***7i***

ADP-ribosyl-N_3_ (0.01 mmol), terminal alkynes (0.012 mmol), 10 mol% CuSO_4_ and Cu(0) (copper sheet) were added in 0.1 M TEAB buffer (1 mL) and stirred for 24 h at room temperature. The reaction was monitored by RP-HPLC analysis. After the reaction was completed, the products were isolated by HPLC chromatogram in a linear gradient of 0–85% of CH_3_CN in water containing 0.1 M TEAB over 50 min on a semipreparative C18-bonded silica column (9.4 mm × 250 mm). After freeze-drying, the products **7a** to **7i** were given in the yields from 79% to 91%. The data for the characterization of compounds **7a**–**7i** were put in the Supplementary Materials of this paper.

### 3.7. Synthesis of ADP-ribosylated Peptide ***9***

ADP-ribosyl-N_3_ (2 mg, 1.2 equiv.), peptide 8 (2 mg), CuSO_4_ (0.75 μM) and Cu(0) (copper sheet) were added in pH = 7.5 PBS buffer (500 μL) and stirred for 24 h at room temperature. The reaction was monitored by analytical RP-HPLC chromatogram in a linear gradient of 10–85% of CH_3_CN in water containing 0.1% TFA over 20 min on a C18-bonded silica column. After the reaction was completed, the products were isolated by HPLC chromatogram in a linear gradient of 10–85% of CH_3_CN in water containing 0.1% TFA over 50 min on a semipreparative C18-bonded silica column (9.4 mm × 250 mm). Compound **9** (2.3 mg, 81%) was obtained after lyophilization. ^1^H-NMR (600 MHz, D_2_O): δ 8.54 (s, 1H), 8.30 (s, 1H), 7.98 (s, 1H), 6.99 (d, *J* = 7.4 Hz, 2H), 6.66 (d, *J* = 7.4 Hz, 2H), 6.31 (s, 1H), 6.06 (s, 1H), 4.66 (s, 1H), 4.60 (s, 1H), 4.53–4.47 (m, 2H), 4.45 (s, 2H), 4.37 (s, 1H), 4.32 (s, 1H), 4.24–4.14 (m, 4H), 4.10 (s, 2H), 4.05 (s, 1H), 3.86–3.77 (m, 3H), 3.72 (m, 1H), 3.13–2.95 (m, 6H), 2.90–2.82 (m, 1H), 2.30 (s, 2H), 1.92 (s, 3H), 1.50 (s, 2H), 1.29 (m, 3H), 1.19 (m, 3H). ^31^P-NMR (162 MHz, D_2_O): δ −11.59. HRMS (ESI) *m*/*z* calculate for ([M + H]^+^): C_49_H_72_N_19_O_24_P_2_^+^: 1372.4467, Found: 1372.4469.

## 4. Conclusions

In conclusion, we described a concise chemical synthesis of ADP-ribosyl-N_3_ and its successful applications for preparations of various ADP-ribosylated molecules. With ribosyl azide as the starting material, ADP-ribosyl-N_3_ could be synthesized through a five-step route including phosphorylation, pryphosphorylation and deprotection reactions. Under the optimized Cu(II)/Cu(0) catalytic system, ADP-ribosyl-N_3_ not only could react smoothly with small molecular alkynes to generate novel dinucleotides in good yields, but also could be conjugated to the free peptide bearing terminal alkyne to produce ADP-ribosylated peptide efficiently. Taking the advantages of synthetic accessibility, moderate stability and desirable reactivity, ADP-ribosyl-N_3_ offered a desirable synthetic precursor for divergent preparations of structurally diverse ADP-ribosylated compounds. The further development of bioconjugation methods in the aid of ADP-ribosyl-N_3_ is undergoing in our lab.

## Figures and Tables

**Table 1:** Screenings and optimizations of reaction conditions.

Entry ^1^	Cu Catalyst	Solvent	Temp.	Yield ^2^
1	CuSO_4_ (10 mol%), sodium ascorbate (5.0 equvi.)	H_2_O	25 °C	<5%
2	CuSO_4_ (10 mol%), sodium ascorbate (5.0 equvi.), TBTA (40 mol%)	H_2_O	25 °C	<5%
3	CuSO_4_ (10 mol%), sodium ascorbate (5.0 equvi.), THPTA (40 mol%)	H_2_O	25 °C	<5%
4	CuI (10 mol%), TBTA (40 mol%)	H_2_O/*t*-BuOH	25 °C	<5%
5	Cu(CH_3_CN)_2_BF_4_ (10 mol%), TBTA (40 mol%)	H_2_O/*t*-BuOH	25 °C	<5%
6	CuSO_4_ (10 mol%), sodium ascorbate (5.0 equvi.)	H_2_O	50 °C	10%
7	CuSO_4_ (10 mol%), sodium ascorbate (5.0 equvi.), TBTA (40 mol%)	H_2_O/*t*-BuOH	50 °C	25%
8	CuSO_4_ (10 mol%), sodium ascorbate (5.0 equvi.), TBTA (40 mol%)	H_2_O/CH_3_CN	50 °C	30%
9	CuSO_4_ (10 mol%), copper sheet, TBTA (40 mol%)	H_2_O/CH_3_CN	50 °C	89%
10	CuSO_4_ (10 mol%), copper sheet	H_2_O/CH_3_CN	50 °C	85%
11	CuSO_4_ (10 mol%), copper sheet	H_2_O/CH_3_CN	25 °C	91%
12	CuSO_4_ (5 mol%), copper sheet	H_2_O/CH_3_CN	25 °C	82%
13	CuSO_4_ (1 mol%), copper sheet	H_2_O/CH_3_CN	25 °C	55%
14	CuSO_4_ (10 mol%), copper sheet	H_2_O/*t*-BuOH	25 °C	88%
15	CuSO_4_ (10 mol%), copper sheet	PBS buffer	25 °C	90%
16	CuSO_4_ (10 mol%), copper sheet	TEAB buffer	25 °C	91%

^1^ ADP-ribosyl-N_3_ (0.1 mmol) and phenylacetylene (0.12 mmol) in 1.0 mL solvent was stirred at a certain temperature for 24 h; ^2^ Isolated yields by semiprepartive HPLC.

## Supplementary Materials

The detailed experimental procedures for the synthesis of starting materials and copies of NMR spectrum of compounds **6**, **7** and **9** are available online.

## Abbreviations

ADP: adenosine diphosphate; NAD^+^: Nicotinamide adenine dinucleotide; AMP: adenosine monophosphate; DIC: *N*,*N*-diisopropylcarbodiimide; TBTA: Tris[(1-benzyl-1*H*-1,2,3-triazol-4-yl)methyl]amine; TEAB: triethylammonium bicarbonate; THPTA: Tris(3-hydroxypropyltriazolylmethyl)amine; CuAAC: copper-catalyzed alkyne and azide cycloaddition.
