# Supplementary material for: ADP-ribosyl-N3: A Versatile Precursor for Divergent Syntheses of ADP-ribosylated Compounds
Source: Molecules. 2017 Aug 14;22(8):1346. doi: 10.3390/molecules22081346 (PMC6152188; doi:10.3390/molecules22081346)
Supplement: Supplementary file 1 [file molecules-22-01346-s001.pdf]

Supporting Information

ADP-ribosyl-N_3: A Versatile Precursor for Divergent Syntheses of ADP-ribosylated Compounds

Lingjun Li*, Qianqian Li, Shengqiang Ding, Pengyang Xin, Yuqin Zhang, Shenlong Huang and Guisheng Zhang*

Collaborative Innovation Center of Henan Province for Green Manufacturing of Fine Chemicals, Key Laboratory of Green Chemical Media and Reactions, Ministry of Education, School of Chemistry and Chemical Engineering, Henan Normal University, Xinxiang, Henan 453007, P. R. China.

The terminal alkynes **a** to **i** used in the CuAAC reaction..S2

Characterization of compound **7a-7l** and **9**…………….…………...…...……S3-S4

Copies of ^{1}H NMR, ^{31}P NMR and ^{13}C NMR spectrums for compound **6**…...........…S5

Compound **6** ^{1}H-^{1}H COSYS...S6

Copies of ^{1}H NMR and ^{31}P NMR spectrums for compound **7a** to **7i**..........……S7-S11

Copies of ^{1}H NMR and ^{31}P NMR spectrums for compound **9**…………..……....…S11

Reference…………………………………………………………...…………S12

Table 1. The terminal alkynes **a** to **i** used in the CuAAC reaction.

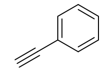

a

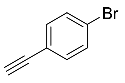

b

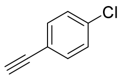

c

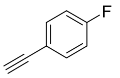

d

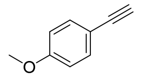

e

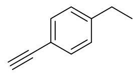

f

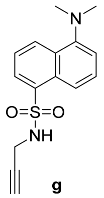

g

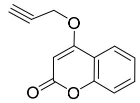

h

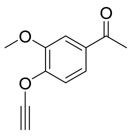

i

Alkyne (**a**, **b, c, d, e, f**), $CuSO_4{\cdot}5H_2O$,Sodium ascorbate and copper(0) from Alfa Aesar. Alkynes (**g, h,** and **i**) was prepared with the method used in our previous work. Alkyne **h**, **g** prepared with the method used in our previous work[1]. The compound of **i** were prepared with the following procedure: 4-hydroxy-3-methoxyacetophenone (1 mmol) was added to a stirred suspension of the bromopropyne (1.0 mmol), potassium carbonate (1mmol) in methanol (5 mL). Upon completion of the reaction (TLC), the mixture was concentrated. The crude mixture was purified by silica gel column chromatography using Ethyl acetate/ petroleum ether as eluent to give target compound.

Characterization of compound 7a-7i:

Compound 7a ^{1}H NMR (400 MHz, D_2O) δ 8.38 (s, 1H), 8.03 (s, 1H), 7.59 (d, *J* = 7.0 Hz, 2H), 7.42 – 7.29 (m, 3H), 6.12 (d, *J* = 4.7 Hz, 1H), 5.83 (s, 1H), 4.66 (s, 1H), 4.51-4.56(m, 2H) 4.43 (s, 2H), 4.32 (m, 1H), 4.32-4.18(m, 4H).^{31}P NMR (162 MHz, D_2O): δ -11.55. HRMS (ESI) m/z calculate for([M+H]$^+$):$C_{23}H_{29}N_8 0_{13}P_2^+$:687.1324, Found: 687.1326.

Compound 7b ^{1}H NMR (600 MHz, D_2O) δ 8.27 (s, 1H), 8.12 (s, 1H), 7.86 (s, 1H), 7.55 (d, *J* = 7.8 Hz, 2H), 7.42 (d, *J* = 7.8 Hz, 2H), 6.44 (d, *J* = 4.8 Hz, 1H), 5.84 (d, *J* = 5.0 Hz, 1H), 4.64 (t, *J* = 4.7 Hz, 1H), 4.57 (s, 1H), 4.50 (d, *J* = 4.7 Hz, 2H), 4.44 (s, 1H), 4.36 (s, 2H), 4.28 (s, 2H), 4.16-4.14 (m, 1H).^{31}P NMR (162 MHz, D_2O): δ -11.71. HRMS (ESI) m/z calculate for ([M+H]$^+$): $C_{23}H_{28}BrN_8O_{13}P_2^+$: 765.0429, Found: 765.0431.

Compound 7c ^{1}H NMR (600 MHz, D_2O) δ 8.40 (s, 1H), 8.20 (s, 1H), 8.01 (s, 1H), 7.52-7.51 (m, 2H), 7.31-7.30 (m, 2H), 6.15-6.14 (m, 1H), 5.79-5.78 (M, 1H), 4.65 (s, 1H), 4.51 (s, 2H), 4.44 (s, 1H), 4.40 (s, 1H), 4.32 – 4.30 (m, 4H), 4.20-4.18 (m, 1H). ^{31}P NMR (162 MHz, D_2O): δ -11.83. HRMS (ESI) m/z calculate for ([M+H]$^+$): $C_{23}H_{28}ClN_8O_{13}P_2^+$: 721.0934, Found: 721.0936.

Compound 7d ^{1}H NMR (600 MHz, D_2O) δ 8.32 (s, 1H), 8.05 (s, 1H), 7.85 (s, 1H), 7.55 – 7.53 (m, 2H), 7.17-7.14 (m, 2H), 6.42 (d, *J* = 4.6 Hz, 1H), 5.88 (d, *J* = 5.0 Hz, 1H), 4.63 (t, *J* = 4.7 Hz, 1H), 4.56 (s, 1H), 4.53-4.52 (m, 1H), 4.49-4.16 (m, 2H), 4.39 – 4.31 (m, 2H), 4.28 (s, 2H), 4.16-4.14 (m, 1H).^{31}P NMR (162 MHz, D_2O): δ -11.74. HRMS (ESI) m/z calculate for ([M+H]$^+$): $C_{23}H_{28}FN_8O_{13}P_2^+$:705.1230, Found: 705.1231.

Compound 7e ^{1}H NMR (600 MHz, D_2O) δ 8.32 (s, 1H), 8.03 (s, 1H), 7.86 (s, 1H), 7.47 (d, *J* = 7.9 Hz, 2H), 6.98 (d, *J* = 7.9 Hz, 2H), 6.42 (d, *J* = 4.2 Hz, 1H), 5.86 (d, *J* = 4.6 Hz, 1H), 4.63 (s, 1H), 4.56 (s, 1H), 4.53 – 4.47 (m, 2H), 4.45 (s, 1H), 4.36-4.33 (m, 2H), 4.28 (s, 2H), 4.16-4.14 (m, 1H), 3.89 (s, 3H).^{31}P NMR (162 MHz, D_2O): δ -11.24. HRMS (ESI) m/z calculate for ([M+H]$^+$): $C_{24}H_{31}N_8O_{14}P_2^+$:717.1429, Found: 717.1431.

Compound 7f ^{1}H NMR (600 MHz, D_2O) δ 8.35 (s, 1H), 8.21 (s, 1H), 7.98 (s, 1H), 7.49 (d, J = 7.7 Hz, 2H), 7.20 (d, J = 7.5 Hz, 2H), 6.12 (d, J = 4.9 Hz, 1H), 5.78 (d, J = 4.8 Hz, 1H), 4.65 (s, 1H), 4.52 (s, 1H), 4.48 (s, 1H), 4.43 (s, 1H), 4.39 (s, 1H), 4.31 – 4.23 (m, 4H), 4.19-4.17(m, 1H), 2.63-2.59 (m, 2H), 1.34 – 1.24 (m, 3H). ^{31}P NMR (162 MHz, D_2O): δ -11.24. HRMS (ESI) m/z calculate for ($[M+H]^+$): $C_{25}H_{31}N_8O_{13}P_2^+$: 715.1637, Found: 715.1639.

Compound 7g ^{1}H NMR (600 MHz, D_2O) δ 8.45 (s, 1H), 8.31-8.32 (m, 1H), 8.18-8.17 (m, 1H), 8.14-8.12 (m, 2H), 7.57 (s, 2H), 7.29-7.25(m, 1H), 6.06 (s, 1H), 5.90 (s, 1H), 4.52 (s, 1H), 4.43-4.39 (m, 2H), 4.30-4.29 (m, 3H), 4.17 (s, 2H), 4.11-4.06 (m, 2H), 3.28-3.25(m, 6H).^{31}P NMR (162 MHz, D_2O): δ -11.55. HRMS (ESI) m/z calculate for ($[M+H]^+$): $C_{30}H_{39}N_{10}O_{15}P_2S^+$: 873.1787, Found: 873.1790

Compound 7h ^{1}H NMR (600 MHz, D_2O) δ 8.32 (s, 1H), 7.72-7.67 (m, 2H), 7.56 (s, 1H), 7.37-7.36 (m, 1H), 7.33 (s, 1H), 7.21-7.20 (m, 1H), 6.50 (s, 1H), 5.94 (s, 1H), 5.90 (s, 1H), 5.30 (s, 2H), 4.60 (s, 1H), 4.55 (s, 1H), 4.47-4.48 (m, 2H), 4.33 (s, 1H), 4.27 (s, 1H), 4.24 (s, 2H), 4.14 (s, 2H).^{31}P NMR (162 MHz, D_2O): δ -11.59. HRMS (ESI) m/z calculate for ($[M+H]^+$): $C_{27}H_{31}N_8O_{16}P_2^+$: 785.1328, Found:785.1329.

Compound 7i ^{1}H NMR (400 MHz, D_2O): δ 8.39 (s, 1H), 8.20 (s, 1H),8.10(s, 1H), 7.66-7.64(m, 1H), 6.01-5.99 (m, 1H), 5.24 (s, 2H), 4.66-4.64 (m, 2H), 4.53–4.49 (m, 2H), 4.45-4.42 (m, 1H), 4.36 (s, 1H), 4.28–4.24 (m, 3H), 4.12-4.11 (m, 1H), 3.91-3.87 (m, 3H), 2.64-2.62 (m, 3H). ^{31}P NMR (162 MHz, D_2O): δ -11.56. HRMS (ESI) m/z calculate for ($[M+H]^+$): $C_{27}H_{35}N_8O_{16}P_2^+$: 789.1641, Found: 789.1642.

Compound **6** ^{1}H, ^{31}P and ^{13}C NMR

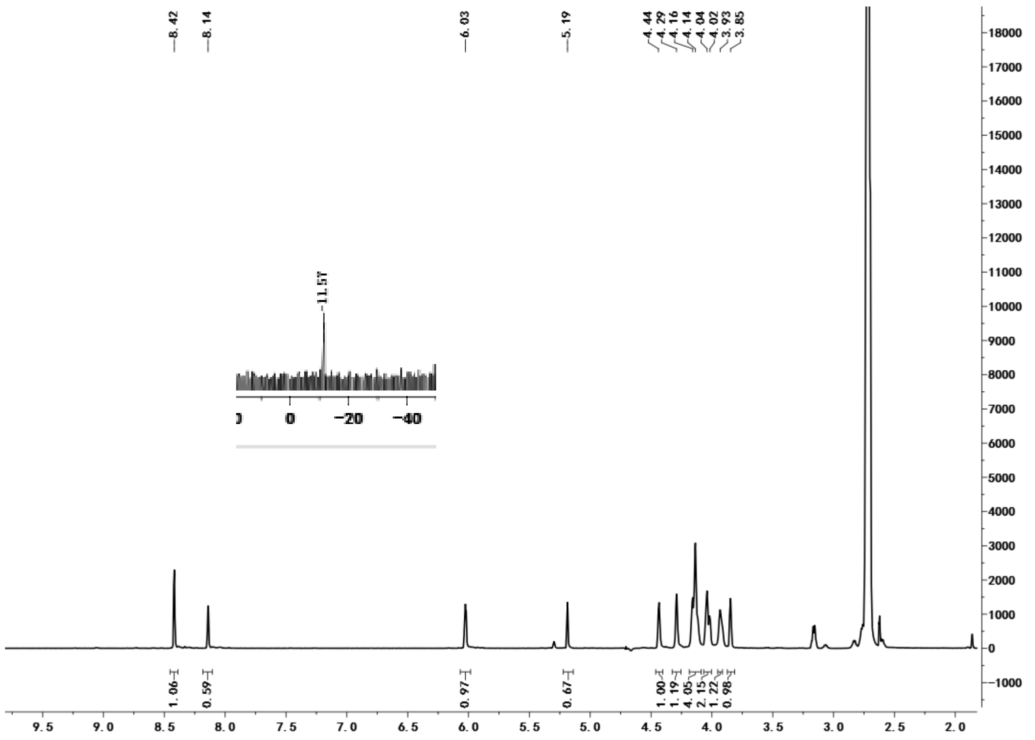

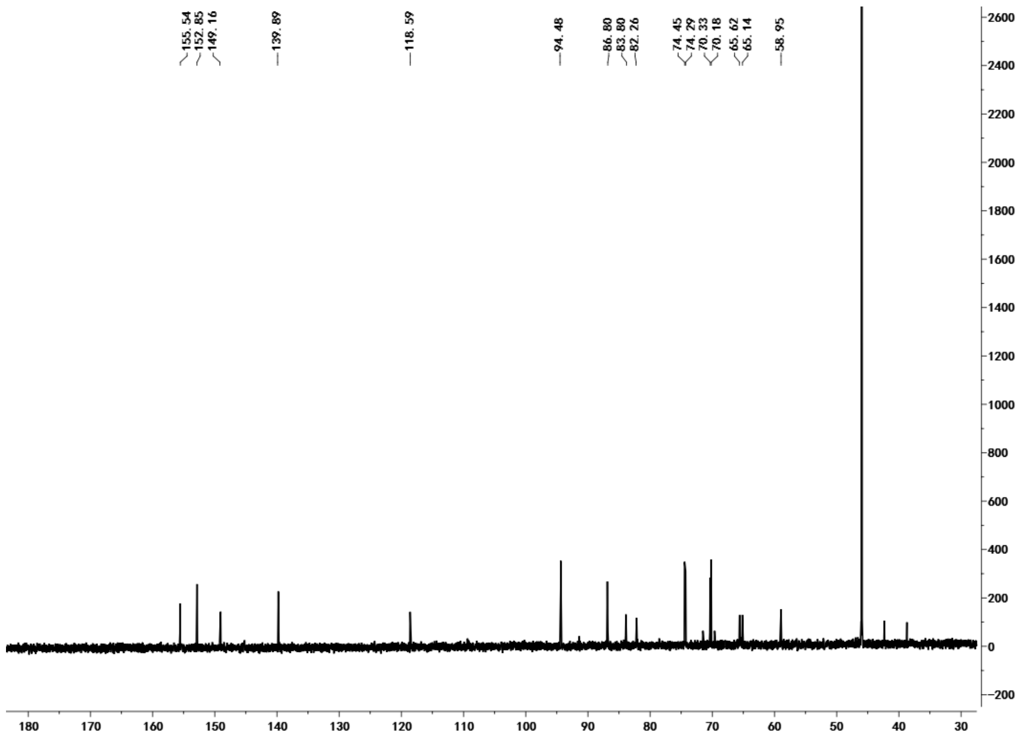

Compound **6** ^{1}H-^{1}H COSY

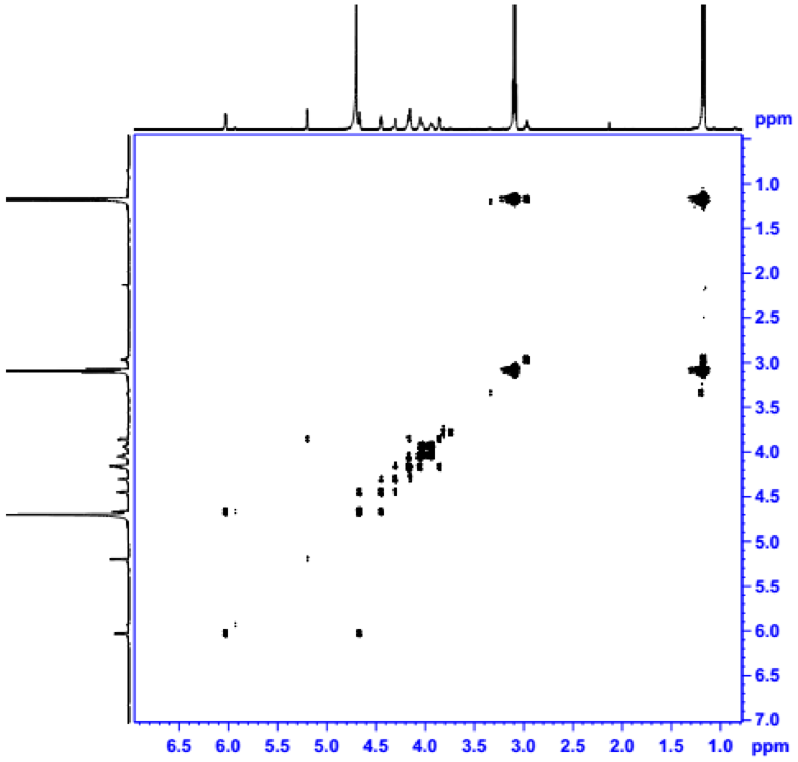

Compound **7a** ^{1}H and ^{31}P NMR

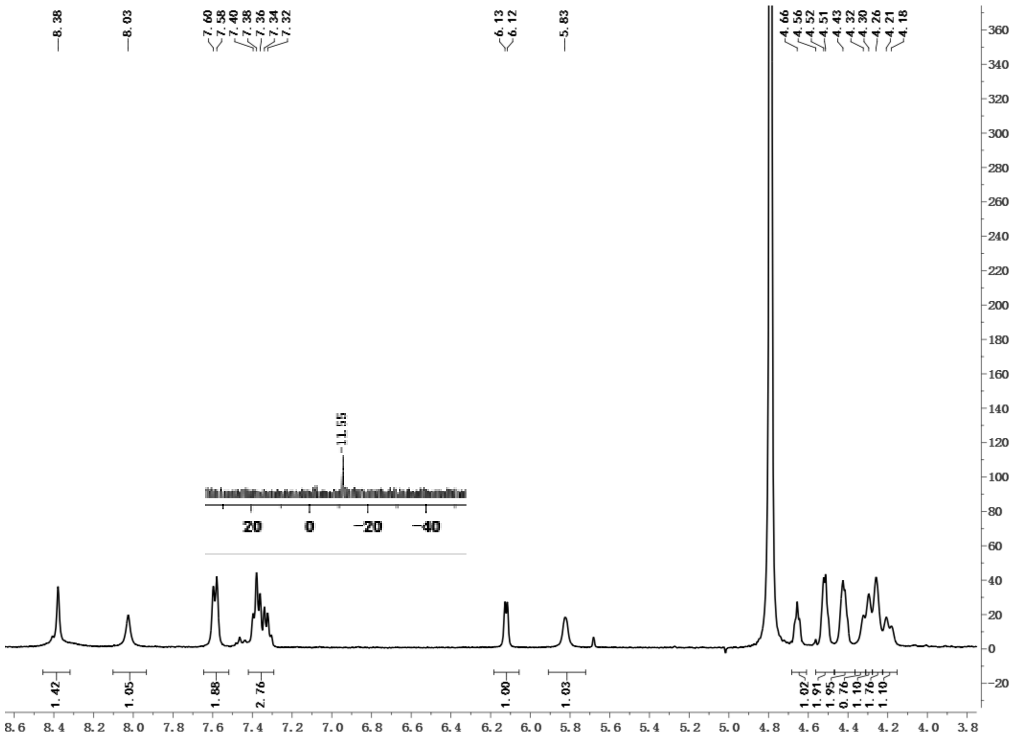

Compound **7b** ^{1}H and ^{31}P NMR

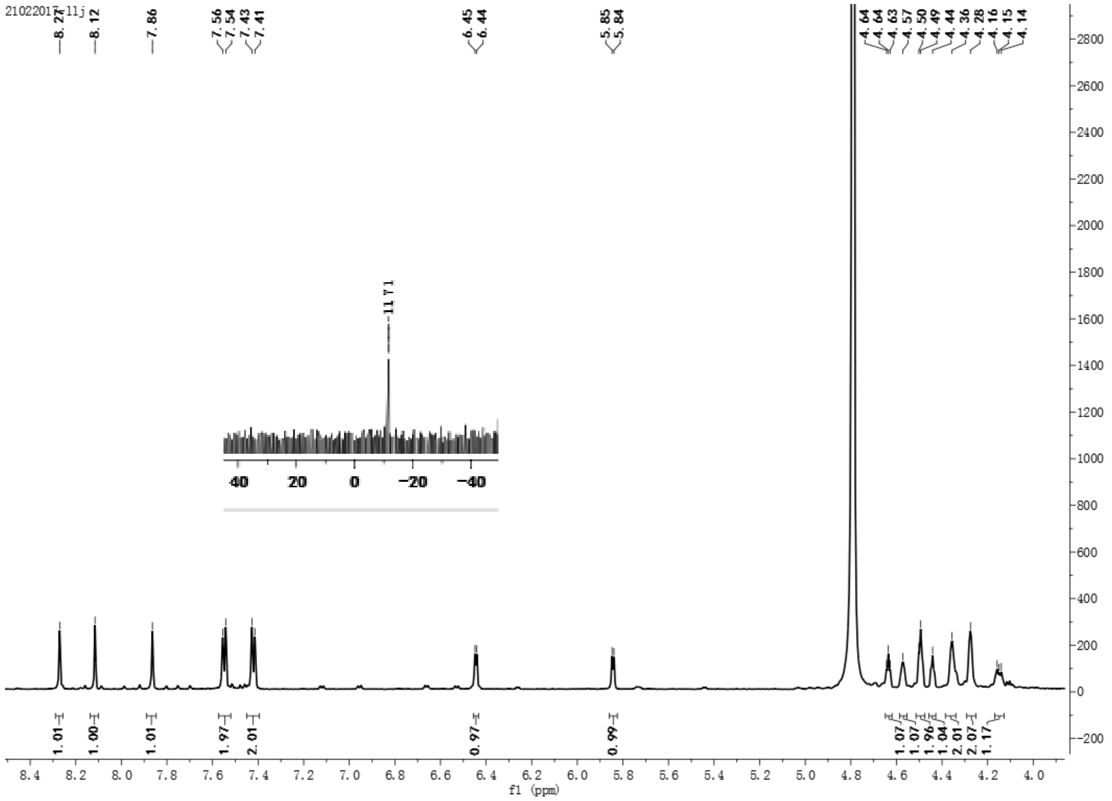

Compound **7c** ^{1}H and ^{31}P NMR

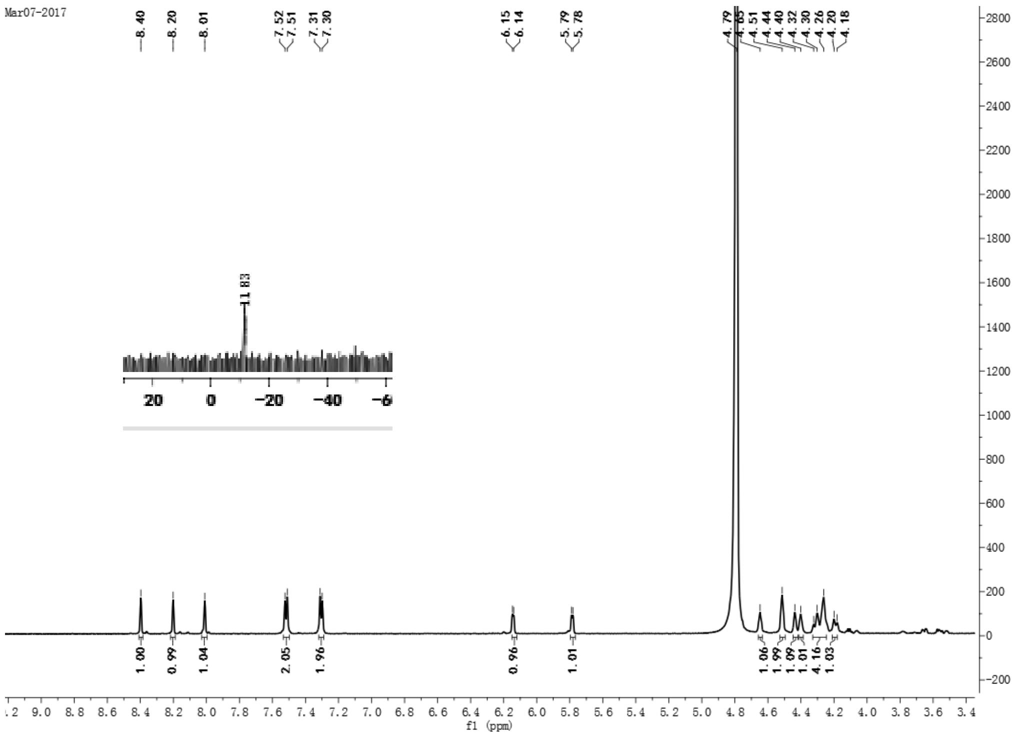

Compound **7d** ^{1}H and ^{31}P NMR

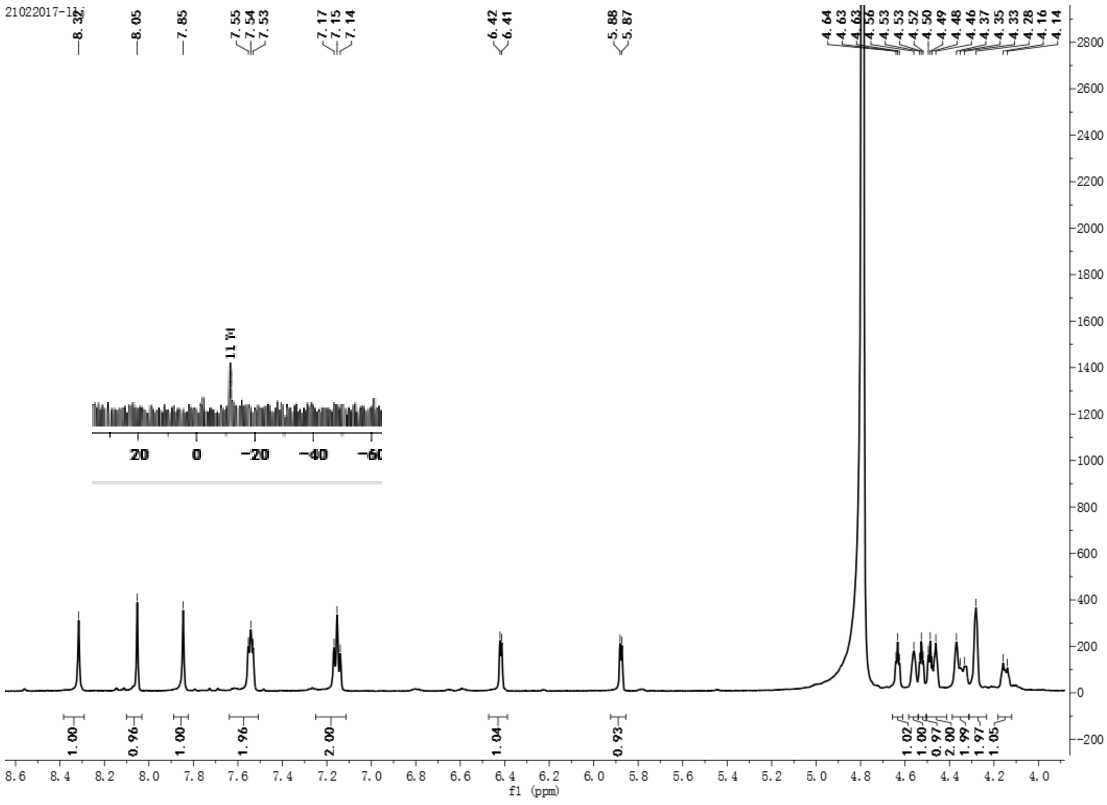

Compound **7e** ^{1}H and ^{31}P NMR

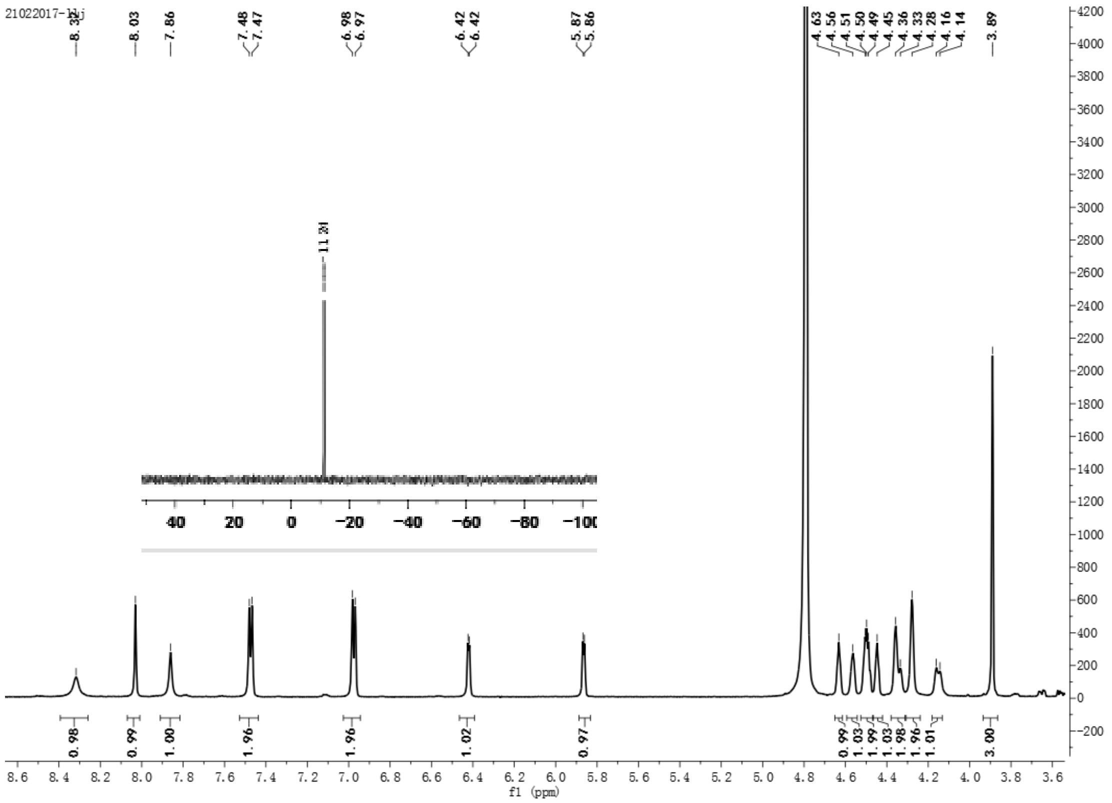

Compound **7f** ^{1}H and ^{31}P NMR

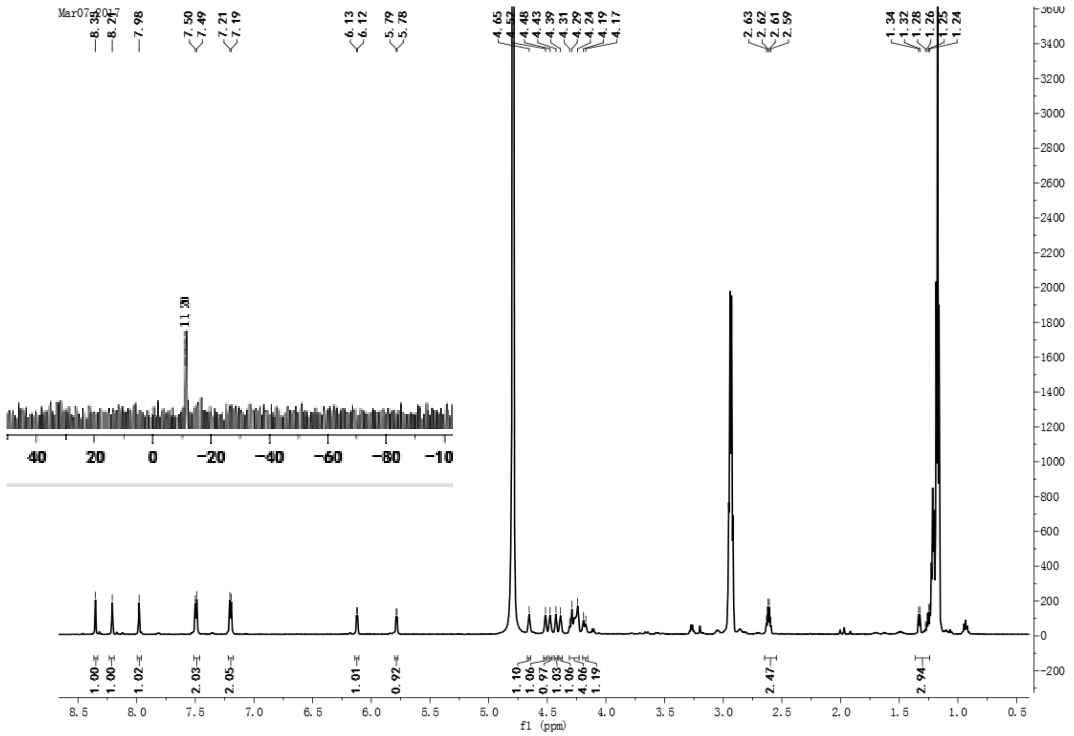

Compound **7g** ^{1}H and ^{31}P NMR

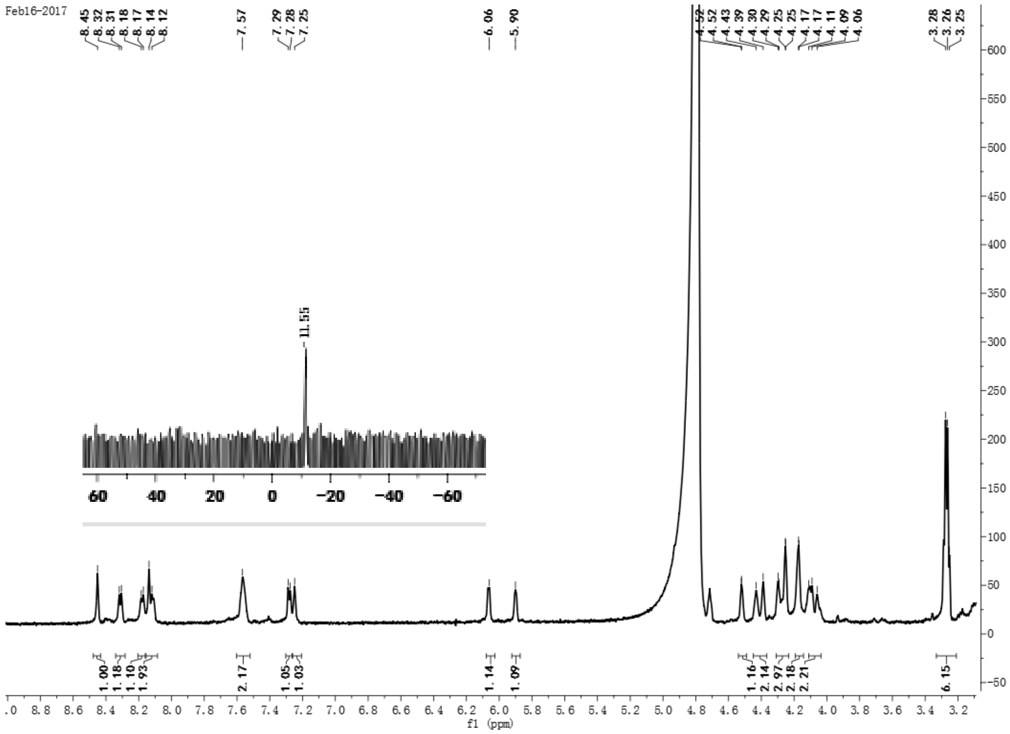

Compound **7h** ^{1}H and ^{31}P NMR

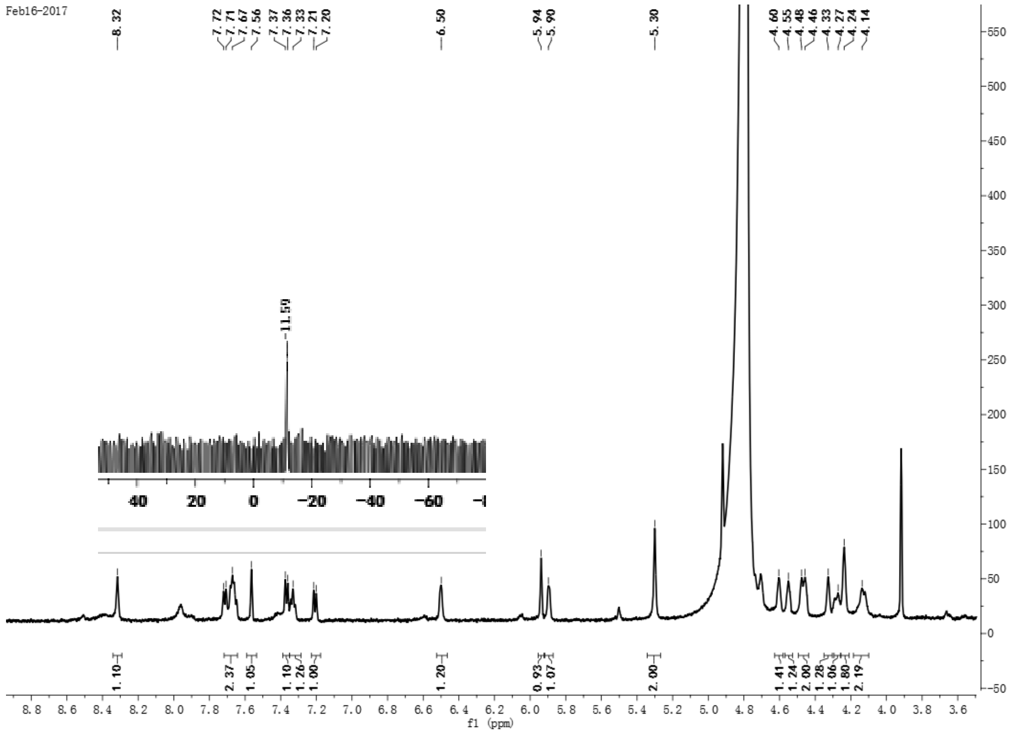

Compound **7i** ^{1}H and ^{31}P NMR

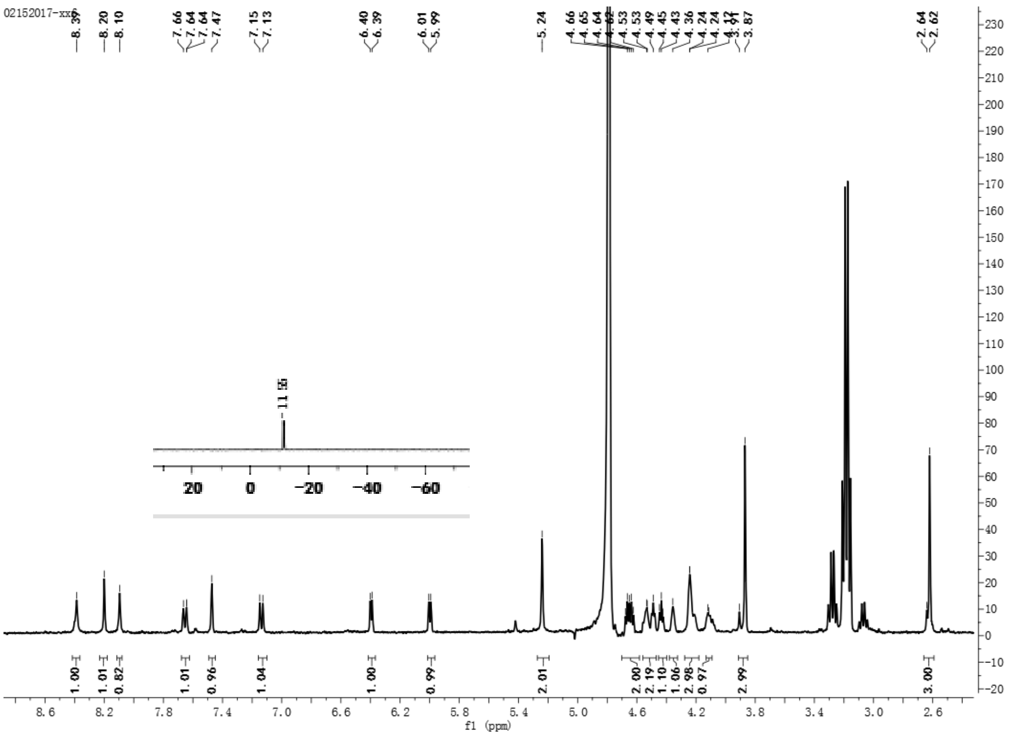

Compound **9** ^{1}H NMR and ^{31}P NMR

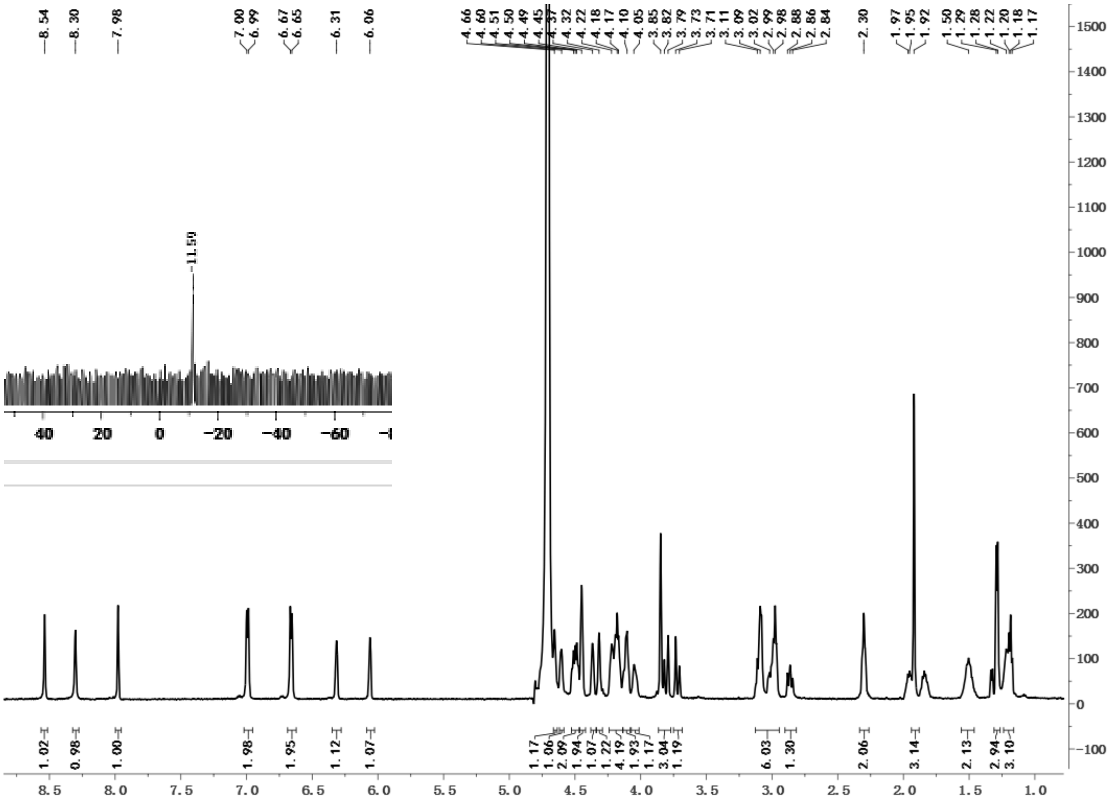

Reference

[1] L. Li, S. Ding, Y. Yang, A. Zhu, *Chem.Eur.J.*, **2017**, *23*, 1166-1172.
